# Safety and Persistence of Nalmefene Treatment for Alcohol Dependence. Results from Two Post-authorisation Safety Studies

**DOI:** 10.1093/alcalc/agab045

**Published:** 2021-07-01

**Authors:** Jonathan Chick, Frank Andersohn, Sylvie Guillo, Kathrin Borchert, Massoud Toussi, Sebastian Braun, Jennifer S Haas, Kavitha Kuppan, Ole M Lemming, Elin Heldbo Reines, Florence Tubach

**Affiliations:** Castle Craig Hospital, Peeblesshire, UK; Frank Andersohn Consulting and Research Services, Mandelstr. 16, 10409 Berlin, Germany; INSERM, Institut Pierre Louis d’Epidémiologie et de Santé Publique, AP-HP, Hôpital Pitié Salpêtrière, Sorbonne Université, 27 rue Chaligny, 75012 Paris, France; Département de Santé Publique, Centre de Pharmacoépidémiologie (Cephepi), CIC-1901, F75013, Paris, France; Xcenda GmbH, Lange Laube 31, 30159 Hannover, Germany; IQVIA, Tour D2, 17 bis Place des Reflets, 92400 Courbevoie, France; Xcenda GmbH, Lange Laube 31, 30159 Hannover, Germany; Xcenda GmbH, Lange Laube 31, 30159 Hannover, Germany; H. Lundbeck A/S, Ottiliavej 9, 2500 Valby, Denmark; H. Lundbeck A/S, Ottiliavej 9, 2500 Valby, Denmark; H. Lundbeck A/S, Ottiliavej 9, 2500 Valby, Denmark; INSERM, Institut Pierre Louis d’Epidémiologie et de Santé Publique, AP-HP, Hôpital Pitié Salpêtrière, Sorbonne Université, 27 rue Chaligny, 75012 Paris, France; Département de Santé Publique, Centre de Pharmacoépidémiologie (Cephepi), CIC-1901, F75013, Paris, France

## Abstract

**Aims:**

Two post-authorisation studies assessed the safety and persistence of patients’ use of nalmefene.

**Methods:**

The START study (EUPAS5678) was a non-interventional, multi-country, prospective, 18-month (8 follow-up visits) cohort study including outpatients initiating nalmefene for the first time. The multi-database retrospective cohort study (MDRC, EUPAS14083) included baseline and follow-up data from German, Swedish and UK healthcare databases. Both studies permitted ‘all comers’ without explicit exclusion criteria; predefined subgroups of interest included the elderly (≥65 years) as well as patients with significant psychiatric and/or somatic comorbidities.

**Results:**

START study: Overall, the mean duration of nalmefene treatment was 10.3 ± 7.3 months (*N* = 1348), with 49.0% of patients treated for ≥1 year; frequent reasons for treatment discontinuation were ‘goal reached’ and ‘drug cost’. The most frequently reported adverse drug reactions (ADRs) were nausea (4.7%), dizziness (3.2%) and insomnia (2.0%). ADR rates appeared higher in the elderly subpopulation (18.6% reported ≥1 ADR vs. 12.0% in the total population) but were not higher in the other predefined subgroups.

MDRC study: The database follow-up analysis followed 2892 patients over 18 months for whom the duration of nalmefene treatment was between 2 and 3 months and <5% of patients used nalmefene for ≥1 year.

**Conclusions:**

Despite the inclusion of a wider patient population (e.g. elderly patients and those with relevant co-morbidities), the safety and tolerability profile of nalmefene given in routine practice was consistent with previous clinical studies. The differing rates of persistence beyond 1 year likely reflect the different methodologies and highlight the relevance of psychosocial support at follow-up visits.

## Teaser

**Short Summary:** Two studies were used to evaluate the safety and persistence of nalmefene in the routine management of alcohol dependence in Europe as part of the overall risk management plan. Although adverse events appeared more common in over 65-year olds, there were no major differences in safety outcomes between the total population, and those with psychiatric and somatic comorbidities or those with a history of seizures. Findings were in line with those from previous clinical studies. The differing rates of persistence beyond 1 year (49% in the prospective study and < 5% in the multi-database retrospective cohort study) likely reflect the different study methodologies and serve to highlight the relevance of follow-up support.

## INTRODUCTION

Alcohol dependence is a common medical and behavioural disorder with a high probability of a chronic relapsing and progressive course. The amount and pattern of alcohol consumed are key risk factors for poor health outcomes, and a dose-dependent relationship between level of alcohol consumption and disease risk has been shown for most categories of diseases or conditions (such as liver disease, cardiovascular disease, cancer and suicide), with higher alcohol consumption conferring greater risk (Gastfriend *et al*., 2007; Rehm *et al*., 2017). However, people with alcohol dependence do not always view abstinence as an acceptable, desirable or realistic treatment goal (Ambrogne, 2002; Gastfriend *et al*., 2007) and many regard the reduction of alcohol consumption as an acceptable and realistic approach to reducing negative consequences (Saladin and Santa Ana, 2004; Heather *et al*., 2010; Gilburt *et al*., 2015).

Nalmefene (Selincro^®^, H. Lundbeck A/S, Valby, Denmark) is an opioid system modulator licenced in Europe (since 2013) and Japan (since 2019) for the reduction of alcohol consumption in adult patients with alcohol dependence who have a high drinking risk level (DRL; defined as alcohol consumption >60 g/day for men and > 40 g/day for women), without physical withdrawal symptoms and who do not require immediate detoxification (Selincro SmPC, 2018). European approval of nalmefene was based on the results of a package of phase III clinical trials which consistently demonstrated that nalmefene, given on an as-needed basis and together with psychosocial support, significantly reduces the total amount of alcohol consumption and number of heavy drinking days in people with alcohol dependence (Gual *et al*., 2013; Mann *et al*., 2013; van den Brink *et al*., 2013; van den Brink *et al*., 2014; Miyata *et al*., 2019). However, as is typical for phase III of a clinical programme, these studies were performed in a relatively homogenous group of patients. For example, patients with DSM-IV Axis I disorders were excluded from the pivotal trials (Gual *et al*., 2013; Mann *et al*., 2013) and, while older age was not an exclusion criterion, few patients were aged over 65 years old (van den Brink *et al*., 2013). In addition, the primarily specialist setting in the pivotal trials may have improved medication adherence and persistence compared to routine practice. Finally, limited study sample sizes and duration of follow up do not allow the identification of potential rare adverse events or long term off-label use.

As part of the overall risk-management plan (RMP) for nalmefene, we aimed to document the frequency of adverse drug reactions (ADRs) in a larger cohort of patients treated in routine clinical practice, including important subpopulations such as those with comorbid psychiatric illness and the elderly. ‘As needed’ dosing with nalmefene represents a paradigm shift in the use of pharmacotherapy for alcohol dependence but there have been concerns about how long a patient should remain on treatment and that *prn* dosing might result in consumption above the recommended dose. Thus, a second aim was to document the patterns of use of the medication over 18 months. Two different studies were performed to address these aims:

1. A multi-country, prospective, non-interventional cohort study (START study), in which outpatients (including those with significant comorbidities) initiating nalmefene therapy for the first time were followed for 18 months (eight scheduled visits).
2. A multi-database retrospective cohort (MDRC) study using longitudinal electronic medical records or claims databases from Germany, Sweden and the UK. This retrospective design guarantees the absence of selection bias and ensures data are collected from the ‘real-life’ treatment setting (where no follow-up or support parameters are imposed).

## METHODS

### The prospective START study

The START study was a non-interventional, multicentre prospective cohort study including outpatients initiating nalmefene therapy for the first time (the first dose of nalmefene intake could be up to 7 days before study inclusion). Patients were then followed for 18 months. The study was registered with the European Union electronic register of post-authorisation studies (EU PAS, Register number: EUPAS5678) and was conducted in accordance with the International Society for Pharmacoepidemiology Guidelines for Good Pharmacoepidemiology Practice. The protocol received all legal agreements and authorizations from independent ethics committee for each site. In Germany and in some Italian sites, the study was only approved for treatment within the licenced indication. All patients provided written informed consent.

The study was conducted in countries where nalmefene was already available for use: Czech Republic, Denmark, Germany, Greece, France, Italy, Poland, Portugal, Romania, Sweden and the UK. Study sites included mixed types of outpatient settings (i.e. general practitioners and specialists) and were recruited to ensure representation in terms of setting or any characteristic of local relevance. Where available, national lists of physicians (Cegedim, Boulogne-Billancourt) were used as sampling frames.

It was assumed that patients would be treated in accordance with the prescribing information and local guidelines; otherwise, no strict inclusion criteria were defined except that the decision to prescribe nalmefene was to be clearly separated from the decision to include the patient in the study. Study medication was used as commercially available and was not supplied by the sponsor for this study. Investigators were asked to consecutively enrol all patients who consented and met the selection criteria, regardless of other considerations. Reasons for non-consent were also recorded.

A total of eight visits were planned (i.e. baseline visit, 1-month visit, 3-month visit, then five visits every 3 months [± 1 month]), reflecting the medical aspects of the management of alcohol-dependent patients in routine clinical practice typical of the public health sector. Descriptive data were collected via an electronic case report form which captured sociodemographic variables, comorbidities and current treatment. The frequency of nalmefene intake was recorded as was the occurrence of any dosing in excess of the licenced daily dose (i.e. >1 nalmefene tablet in any given day) based on patient’s spontaneous reporting or investigator non-leading questions or observations. The duration of nalmefene treatment was estimated based on the date of treatment discontinuation, and reasons for stopping were collected. Long-term use was defined as nalmefene treatment beyond 1 year. In addition, alcohol consumption (frequency and intensity) at each visit was assessed using the Alcohol Use Disorders Identification Test-Consumption (AUDIT-C).

ADRs (defined as an unintended or noxious response to the medication) were collected following patient spontaneous reporting or investigator non-leading questions/observation. ADR collection started after the first dose of nalmefene intake (which could be up to 7 days before the signature of informed consent) and continued until the final follow-up visit. ADRs of special interest as predefined in the RMP were confusional state, hallucinations, dissociation, convulsions and depression, chosen because they had appeared as treatment-emergent adverse events in previous clinical studies.

For the START study, we estimated that a sample size of 2000 patients was needed to obtain precision of 0.25–1.0% for ADRs of special interest (which affected 0.3 to 3.2% of patients in the pivotal trials) (Gual *et al*., 2013; Mann *et al*., 2013; van den Brink *et al*., 2014). All descriptive analyses were observed case, with no imputation for missing data.

Taking into account the RMP, several subpopulations of interest were predefined at baseline. In this report we focus on five predefined subgroups of interest; other predefined subgroups are reported in Table e1.

1. Elderly (aged ≥65 years old at baseline) patients;
2. Patients with significant psychiatric comorbidities (depressive episode, anxiety disorders, schizophrenia/psychosis, bipolar disorder, eating disorders, sleep disorders and other substance use disorders);
3. Patients with significant somatic comorbidities;
4. Patients with a history of seizure disorder, including alcohol withdrawal seizures, at baseline; and
5. Pregnant or lactating women at any time during the study participation.

### The multi-database retrospective cohort (MDRC) study

This was a retrospective cohort study using longitudinal electronic medical records and administrative claims from automated databases. The study was registered at the EU PAS (EUPAS14083). Three countries were identified as having suitable and complementary databases for the analysis of drug persistence and potential overdosing:

1. German Statutory Health Insurance (SHI) claims database.
2. Swedish Prescribed Drug Register, Swedish Patient Register, Swedish Medical Birth Register and Swedish Cause of Death Register.
3. UK Clinical Practice Research Datalink General Practitioners Online Database.

Separate analyses were performed at baseline and at the end of the 18-month follow-up. Outcomes included demographics, presence of comorbidities, duration of treatment and occasions where a patient was suspected to have taken more than one tablet in a day. Baseline analyses included all patients with an incident prescription/dispensing (first prescription/dispensing) who were prescribed nalmefene between the launch date in each country (Germany and Sweden: 1 September 2014, UK 1 May 2013) and a database-specific end-of-inclusion date (Germany: 30 June 2016; Sweden: 29 April 2017; UK: 28 February 2017). For Sweden, the analyses on comorbidities were restricted to patients who could be linked to the Swedish National Patient Register (SNPR). For the follow-up analyses, the end-of-inclusion date was set to 30 June 2016 and data were collected for 18 months from the first nalmefene prescription.

## RESULTS

### The prospective START study

Of the 22,077 sites approached, 540 sites (2.4%) sent back a feasibility questionnaire and 99 sites were initiated and included patients. Fifteen sites were in primary care and 84 sites provided specialty care (alcohol specialists, psychiatry, gastroenterology, hepatology). A total of 1869 patients were screened for participation, of which 1420 patients were included and followed between 28 August 2014 and 12 March 2019. The ‘Safety population’ included the 1373 patients who took at least one dose of study medication. The total analysis population (TAP) included 1348 patients; key reasons for exclusion from the TAP were: ‘not receiving nalmefene for the first time’ (36.3%), ‘informed consent not provided’ (34.3%), ‘investigator’s decision’ (24.7%) and ‘patient initiated and stopped nalmefene within the 7 days before the baseline’ (4.7%). The number of patients at each designated visit decreased over time, from 1373 at baseline to 920 at Month 18 in the Safety population, and from 1348 at baseline to 909 at Month 18 in the TAP.

Baseline characteristics for the TAP are shown in Table 1. Most (71.7%) were male; the mean ± SD age was 47.9 ± 11.8 years. The mean AUDIT-C total score was 8.4 ± 2.8 at baseline (ranging from 7.3 in Italy to 9.6 in the UK; where a score of ≥5 is considered an increased risk for active alcohol misuse or dependence (Rumpf *et al*., 2002)). Overall, 68.8% of patients in the TAP had at least one comorbidity; 7.6% were aged ≥65 years, and 12.5% had a history of seizure. The prevalence of psychiatric disorders was 55.9% (depressive disorders, 33.2%; anxiety disorders, 18.3%; sleep disorders, 15.4%; other substance use disorders 5.3%; bipolar disorder, 4.6%; schizophrenia/psychosis, 3.2%; eating disorders, 3.0%). The use of concomitant CNS-active medications was similarly high, with 795 (59.0%) of patients in the TAP taking ≥1 CNS active medication. More than a third of patients (37.7%) had significant somatic comorbidities (cardiac/vascular disorders, 19.4%; gastrointestinal/hepatobiliary disorders, 13.2%; nervous system disorders, 7.3%; endocrine disorders, 5.9; respiratory, thoracic and mediastinal disorders, 4.4%).

**Table 1:** Baseline characteristics

	START study	MDRC study		
	TAP *N* = 1348	Germany *N* = 610	Sweden *N* = 2536 (*N* = 1848)^a^	UK *N* = 247
Age (years); mean ± SD	47.9 ± 11.8	48.9 ± 10.4	52.5 ± 13.4	49 ± 11.0
Age (years); *n* (%) <18 18–64 ≥65	0 1245 (92.4%) 103 (7.6%)	0 576 (94.4%) 34 (5.6%)	0 2047 (80.7%) 489 (19.3%)	0 225 (91.1%) 22 (8.9%)
Sex Male Female	967 (71.7%) 381 (28.3%)	368 (60.3%) 242 (39.7%)	1709 (67.4%) 827 (32.6%)	142 (57.5%) 105 (42.5%)
Age (years) at onset of drinking problems; mean ± SD	*N* = 1121 32.6 ± 12.1	NR	NR	NR
Previous formal treatment for alcohol dependence; *n* (%)	884 (65.6%)	NR	NR	NR
Comorbidities; *n* (%)^a^ ≥1 comorbidity ≥1 somatic comorbidity ≥1 psychiatric comorbidity	928 (68.8%) 508 (37.7%) 754 (55.9%)	–482 (79.0%) 507 (83.1%)	–374 (20.2%) 378 (20.5%)	–112 (45.3%) 46 (18.6%)
Concurrent CNS-active medications; *n* (%) Antidepressant Antipsychotics Anxiolytics Hypnotics Opioids	549 (69.1%) 187 (23.5%) 374 (47.0%) 224 (28.2%) 13 (1.6%)	283 (46.4%) 122 (20.0%) 39 (6.4%) 26 (4.3%) 17 (2.8%)	921 (36.3%) 182 (7.2%) 533 (21.0%) 647 (25.5%) 134 (5.3%)	133 (53.8%) 21 (8.5%) 37 (15.0%) 25 (10.1%) 21 (8.5%)

^a^For Sweden, the number of patients who could be linked to the SNPR was *N* = 1848 in the Swedish database. This denominator was used for the evaluation of comorbidities. NR: not reported.

Laboratory samples for ALAT or ASAT (serum alanine aminotransferase, serum aspartate aminotransferase) were available at baseline for 27.8% of patients from the TAP; 28 out of 335 patients (8.4%) had an ASAT value >3-fold ULN, and 18 out of 353 (5.1%) had an ALAT value >3-fold ULN. Two-thirds of patients (*N* = 919, 68.2%) were potentially receiving nalmefene off-label. This number was mainly driven by the number of patients classified as low/medium DRL according to the algorithm derived from AUDIT-C (*N* = 866; 64.2%).

**Fig. 1.**
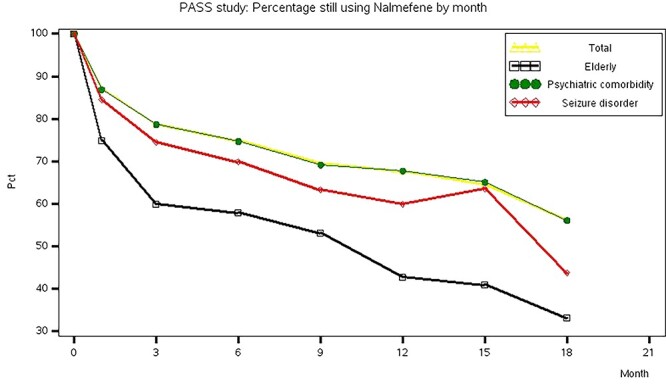
Per cent of patients still using nalmefene at each follow-up visit of the START study; only patients with available information on ongoing nalmefene use at the respective follow-up visit were considered in the calculation of percentages.

In the TAP, the mean duration of nalmefene treatment was 10.3 ± 7.3 months, with 49.0% of patients treated for >1 year (Table 2). The per cent of patients still using nalmefene at each follow-up visit is shown in Fig. 1, where 67.7% of patients were on treatment at 1-year visit (note that the figure percentages only include patients with available information on ongoing nalmefene use at each visit). The mean duration of nalmefene treatment was shorter among elderly patients (8.1 ± 6.9 months) and patients with history of seizure disorder (7.8 ± 7.0 months), and the proportion of patients using nalmefene for ≥1 year was also lower in these subgroups (35.6% of elderly patients and 32.0% of patients with history of seizure disorder vs. 49.0% of patients in the TAP). Patients with a psychiatric comorbidity had a similar duration of treatment to the TAP population (this subgroup accounted for >50% of the TAP).

**Table 2:** Outcomes at end of follow-up

	START study					MDRC study		
	Total population, *N* = 1348	Elderly population, *N* = 103	Significant psychiatric comorbidity, *N* = 714	Significant somatic comorbidity, *N* = 441	Seizure disorder, *N* = 168	Germany, *N* = 494	Sweden, *N* = 2167	UK, *N* = 231
Duration of nalmefene treatment (months); mean ± SD	10.3 ± 7.3	8.1 ± 6.9	10.6 ± 7.3	8.8 ± 7.3	7.8 ± 7.0	3.3 (4.4)	1.80 (2.32)	3.1 (3.9)
Long-term use (>1 year); *n*/*N* (%)	624/1273 (49.0%)	36/101 (35.6%)	348/679 (51.3%)	171/417 (41.0%)	49/153 (32.0%)	20 (4.1%)	34 (1.6%)	7 (3.0%)
Any overdose; *n*/*N* (%)	8/1275 (0.6%)	0	2/681 (0.3%)	2/417 (0.5%)	0	30 (6.1%)	20 (0.9%)	38 (16.5%)
ADR; *n* (%) ≥1 ADR ≥1 ADR of special interest	*N* = 1373 155 (12.0%) 20 (1.5%)	*N* = 104 19 (18.6%) 2 (1.9%)	*N* = 723 90 (13.1%) 10 (1.4%)	*N* = 452 71 (16.7%) 13 (2.9%)	*N* = 172 23 (14.7%) 6 (3.5%)	NR	NR	NR

Across the 18 months of follow-up, the most frequent reasons for treatment discontinuation were ‘goal reached’ and ‘drug cost’ (Table 3). There were nine occasions (8 patients) of a patient taking more than the recommended dose of one tablet per day; seven patients (8 occasions) took two tablets of nalmefene (36 mg) on the same day and one patient took three tablets of nalmefene (54 mg) on the same day.

**Table 3:** Reasons for discontinuation by month (START study, TAP)

	Month 1	Month 3	Month 6	Month 9	Month 12	Month 15	Month 18
Number of patients stopping	40	38	29	37	33	37	352
ADR	19 (47.5%)	10 (26.3%)	6 (20.7%)	6 (16.2%)	7 (21.2%)	2 (5.4%)	28 (8.0%)
Lack of efficacy	4 (10.0%)	5 (13.2%)	9 (31.0%)	5 (13.5%)	6 (18.2%)	2 (5.4%)	30 (8.5%)
Drug cost	2 (5.0%)	4 (10.5%)	3 (10.3%)	5 (13.5%)	3 (9.1%)	7 (18.9%)	105 (29.8%)
Goal reached	9 (22.5%)	5 (13.2%)	3 (10.3%)	9 (24.3%)	12 (36.4%)	19 (51.4%)	133 (37.8%)
Goal changed	0 (0.0%)	2 (5.3%)	1 (3.4%)	1 (2.7%)	1 (3.0%)	1 (2.7%)	15 (4.3%)
Goal abandoned	4 (10.0%)	9 (23.7%)	5 (17.2%)	5 (13.5%)	3 (9.1%)	5 (13.5%)	34 (9.7%)
Other	2 (5.0%)	3 (7.9%)	2 (6.9%)	6 (16.2%)	1 (3.0%)	1 (2.7%)	7 (2.0%)
Missing	–	1	–	1	–	1	–

A total of 155 patients (12.0%) reported at least one ADR (Table 2). The most frequently reported ADRs were nausea (4.7%), dizziness (3.2%), insomnia (2.0%), vomiting (1.7%) and headache (1.5%) (Table 4). Twenty patients (1.5%) reported 23 ADRs of special interest, including seven events of confusional state (including one serious event reported as ‘disorientation’, all resolved), six events of hallucination, five events of dissociation and five events of depression (all five were classified as serious ADR). There was no report of convulsions and no ADR of special interest led to treatment withdrawal or to death. Rates of ADR appeared higher in the elderly subpopulation (18.6% of elderly patients reported ≥1 ADR during follow-up vs. 12.0% in the TAP). Three participant pregnancies were reported during the study period (after 15 and 18 months of follow-up); no data (including ADRs of special interest) were reported in these women. One pregnant woman experienced serious ADRs of spontaneous abortion and a suicide attempt. ADR frequencies in additional subgroups are included in the supplementary material.

**Table 4:** Summary of ADRs reported in the START study compared to reporting in the Nalmefene European Summary of Product Characteristics (SmPC)

	Safety Population *N* = 1373	European Summary of Product Characteristics
ADR occurring in ≥ 2% of patients		
Nausea	64 (4.7%)	Very common (≥10%)
Dizziness	44 (3.2%)	Very common (≥10%)
Insomnia	28 (2.0%)	Very common (≥10%)
ADR of special interest		
≥1 ADR of special interest	20 (1.5%)	
Confusional state	6 (0.4%)	Common (≥1% to <10%)
Depression	5 (0.4%)	NR
Dissociation	3 (0.2%)	Not known
Depersonalisation/derealisation disorder	2 (0.1%)	NR
Derealisation	2 (0.1%)	NR
Hallucination, visual	2 (0.1%)	Not known
Autoscopy	1 (0.1%)	NR
Disorientation	1 (0.1%)	NR
Hallucination	1 (0.1%)	Not known

NR: not reported in the Nalmefene SmPC.

**Fig. 2.**
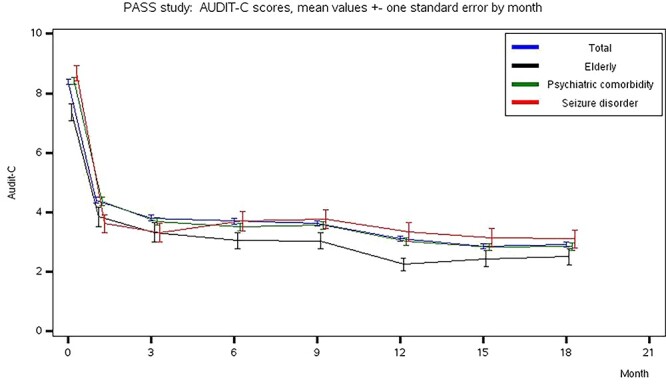
AUDIT-C total scores at each follow-up visit of the START study.

Alcohol consumption as measured by the AUDIT-C questionnaire decreased during follow-up. The mean AUDIT-C total score improved from 8.4 ± 2.8 at baseline to 2.9 ± 3.1 at Month 18 in the TAP and similar trends were observed within the different subgroups of interest (Fig. 2).

### The multi-database retrospective cohort study

The MDRC baseline analysis included 3393 patients treated with nalmefene in the UK (*n* = 247), Germany (*n* = 610) and Sweden (*n* = 2536). For Sweden, the number of patients who could be linked to the SNPR was *N* = 1848. Baseline characteristics per country are given in Table 1. The majority of patients were between 18 and 64 years old; with smaller proportion of elderly users in the UK and Germany compared to Sweden (8.9% and 5.6% vs. 19.3%, respectively). More men than women were prescribed nalmefene and there were high rates of somatic and psychiatric comorbidities reported. Psychiatric comorbidities were more commonly reported for patients in the German SHI database than in the Swedish or UK databases (79.0% vs. 20.2% and 45.3%, respectively). Seizures or alcohol withdrawal state during the past 12 months were recorded in 200 patients in Germany (32.8%), 89 in Sweden (4.8%) and 3 (1.2%) in the UK. Five women (in Germany and Sweden) became pregnant during treatment with nalmefene (with no further data reported). The proportion of patients who were prescribed nalmefene without a recorded diagnosis of alcohol dependence varied between 18.0% in Germany (110 from 610 patients), 51.3% in the UK (121 from 236 patients) and 68.4% in Sweden (1482 from 2167 patients).

The MDRC follow-up analysis included a total of 2892 patients (Germany: *n* = 494; Sweden: *n* = 2167; UK: *n* = 231); 116 patients from Germany were included in the baseline analyses, but not in the follow-up analyses due to an administrative change in the database. Overall, the mean duration of nalmefene treatment during follow-up was between 2 and 3 months (Table 2). The proportion of patients who used nalmefene for ≥1 year was <5% in all three databases: 3.0% (7 patients) in the UK, 4.1% (20 patients) in Germany and 1.6% (34 patients) in Sweden. Most (61.9–84.6%) nalmefene users had only one record of a nalmefene prescription during the observation period. Few patients took more than the recommended dose of one tablet per day, and there were fewer recorded instances of possible ‘overdosing’ (i.e. >1 tablet per day) in Germany and Sweden than in the UK (6.1% and 0.9% vs. 16.5%, respectively).

## DISCUSSION

Results from these routine practice studies provide valuable safety data in important subpopulations of patients who were not the main population studied in the previous pivotal studies (Gual *et al*., 2013; Mann *et al*., 2013; van den Brink *et al*., 2014; Miyata *et al*., 2019) and confirm the favourable benefit–risk profile of nalmefene treatment in the wider alcohol-dependent population. Results show no major differences in safety outcomes between the total population, and those with psychiatric and somatic comorbidities or a history of seizures, except a slightly greater prevalence of ADRs in the elderly. Though limitations of the data sources must be considered, the START and MDRC studies identified no concerns with respect to use of nalmefene over more than 1 year and how often a patient might take more than the recommended dose of one tablet per day.

Despite the inclusion of a wider patient population treated in routine practice, the types of ADR seen with nalmefene were in line with the profile seen in the previous clinical studies (Gual *et al*., 2013; Mann *et al*., 2013; van den Brink *et al*., 2014; Miyata *et al*., 2019), and no new safety concerns were identified. The most commonly reported ADRs were nausea, dizziness and insomnia, which are expected with opioid antagonism and which were typically mild-to-moderate and transient in the pivotal studies (van den Brink *et al*., 2014). Rates of ADR were, in general, lower than rates of adverse events in the pivotal studies, likely reflecting the study setting and also the difference in definitions (whereas ADR focuses on treatment-related events, an adverse event should capture any untoward medical occurrence). In the pivotal studies, the higher incidence of psychiatric adverse events in the nalmefene group versus placebo was mainly due ‘confusional state’ which affected 1.2% of nalmefene patients (vs. 0.3% in the placebo group) and was thus considered an ADR of special interest in the RMP (van den Brink *et al*., 2015). In the START study, confusional state was reported as an ADR for six patients (0.4%) with an additional serious event of disorientation in one patient. Here it is intriguing that no ADR of special interest (including confusional state) led to treatment discontinuation indicating that patients and their doctors decided to carry on or resume treatment despite these ADRs. Indeed, all patients showing a confusional state had complete recovery and no further events of this kind were reported with continued treatment.

Unlike the pivotal studies, these routine practice studies included high proportions of patients with comorbid psychiatric illness, with high levels of concomitant CNS-active medication. The impact of these comorbidities and concomitant treatments are important to explore as alcohol dependence often coexists and often exacerbates other psychiatric disorders (Castillo-Carniglia *et al*., 2019) and current guidelines stipulate an integrated approach to treatment. While we did not analyse the different disorders separately, it is of practical importance that the subgroup with psychiatric comorbidities showed a similar reduction in alcohol consumption (as indicated by AUDIT-C scores) to the overall population and other subgroups. This is relevant as, for example, there is good evidence that reducing alcohol consumption improves mood or anxiety symptoms (Allan *et al*., 2002; Charlet and Heinz, 2017; Gallagher *et al*., 2018). Conversely, meta-analyses of studies that have supplemented treatment for alcohol use disorders with conventional treatments for anxiety and/or depression have only found (at best) a small ‘boost’ in the benefit of AUD treatment outcomes (Torrens *et al*., 2005; Hobbs *et al*., 2011; Kelly *et al*., 2012).

While older individuals often drink less and report fewer alcohol-related problems than younger individuals (Atkinson, 1990), alcohol dependence remains a significant health issue for older patients. Although elderly patients had a higher rate of ADR during follow-up (18.6% of elderly patients vs. 12.0% in the TAP), the generally favourable benefit–risk profile observed for nalmefene in this population is important because there are often certain precautions with other medications for alcohol dependence. For example, it is current practice to avoid using disulfiram in the older population due to the risk of cardiovascular side effects, medication interactions and exacerbation of underlying medical conditions (Kuerbis and Sacco, 2013; Kok, 2014). While there is some evidence for naltrexone in the older population (Oslin *et al*., 1997), nalmefene may be preferred because there is no need to monitor liver function before and during treatment.

Prescription patterns for nalmefene showed that many patients discontinue treatment over the course of 1 year, with much higher rates of patient retention in the prospective START study than recorded in the MDRC study. In the START study, the most common reason for stopping treatment was ‘goals reached’, which is a positive outcome for the patient. In the MDRC study, the mean duration of nalmefene treatment was between 2 and 3 months and the proportion of patients who used nalmefene for ≥1 year was <5%. This is in line with the time course of alcohol reduction with the as needed use of nalmefene which, in the pivotal studies, showed significant alcohol reduction as early as 1 month that was maintained through the 6-month treatment periods (Gual *et al*., 2013; Mann *et al*., 2013). The higher retention rates in the START study versus MDRC likely reflects the differences in the methodologies employed. While a prospective study is preferable for collecting ADR data, the key advantage of using a retrospective database study design for persistence data is that it guarantees the absence of selection bias and a routine environment, including a routine level of psychosocial support. Just being included in a clinical study may have enhanced START study persistence outcomes, particularly as patients had to attend eight visits in 18 months to regularly discuss their alcohol intake.

Strengths of our analyses include the size of the international samples studied and the inclusion of important subgroups of patients, who are commonly encountered in clinical practice, but were excluded from the pivotal studies. Limitations include the fact we did not reach our intended sample of 2000 patients in the START study, although the reduction in precision for ADRs of special interest with 1420 patients was marginal. Of >22,000 sites contacted for the START study, only 2% were interested in participating. These difficulties may be explained by the limited market access conditions in some countries. For example, in the UK, restrictions on primary care prescribing of nalmefene were a key barrier to UK site recruitment in the START study. Using AUDIT data to approximate alcohol consumption and DRL, it appears that 68.2% of patients in the START study potentially had a low/medium DRL. While this may potentially limit the generalisability of our findings to a less severely affected population (many of whom would not qualify for treatment under the nalmefene label), such data should be interpreted with caution since the study did not capture prior history (unlike the pivotal trials which largely comprised new patients seeking treatment for the first time, 65.6% of START study patients had a prior history of formal treatment for alcohol dependence) as well as other missing data. It is also important to note that the AUDIT-C was used as a pragmatic, easy-to-use measure of consumption that approximates current practice even though it was developed as a screening tool to identify patients with hazardous or harmful alcohol use and not as a tool for estimating alcohol consumption (Bradley *et al*., 2007). Moreover, the AUDIT observations cannot replace the repeated findings in the pivotal trials of nalmefene showing that positive effects are mainly restricted to patients with high/very high DRL at baseline (Gual *et al*., 2013; Mann *et al*., 2013). Although the START study is one of the first studies to evaluate nalmefene use in important subgroups, the categories were somewhat broad, and we did not look at each specific comorbidity separately. For example, the somatic subgroup included patients with cardiovascular and gastrointestinal disorders as well as cancer, each associated with a myriad of comorbidities and concomitant medications. Treatment outcomes in more homogenous populations of people living with specific comorbidities merits further work.

In summary, our findings support and extend previous observational studies and randomized clinical trials and suggest that nalmefene is associated with reduced alcohol use in real-world settings where there are frequent comorbidities and concomitant medications. No new safety signals were detected overall and for any of the subpopulations of interest.

## Supplementary Material

Chick_et_al_Supplemental_Appendix_agab045

## Data Availability

Study protocols and datasets are available from the corresponding author on reasonable request.
