# Supplementary material for: Safety and Persistence of Nalmefene Treatment for Alcohol Dependence. Results from Two Post-authorisation Safety Studies
Source: Alcohol Alcohol. 2021 Jul 1;56(5):556–64. doi: 10.1093/alcalc/agab045 (PMC8406067; doi:10.1093/alcalc/agab045)
Supplement: Chick_et_al_Supplemental_Appendix_agab045 [file chick_et_al_supplemental_appendix_agab045.docx]

**Supplemental Appendix**

**Table e1.**

**Proportion of patients in the START study with adverse drug reactions in additional subgroups of interest**

| Adverse drug reaction; n (%) | Patients with concurrent use of other CNS-active medicines N=772 | Patients with long term use of nalmefene (>1 year)  N=633 | Patients with increased ALAT or ASAT*  N=35 | Patients with concurrent use of opioids  N=19 | Other ethnic groups than Caucasian  N=6 | Off-label use N=931 |
| --- | --- | --- | --- | --- | --- | --- |
| ≥1 ADR | 30 (3.9%) | 37 (5.8%) | 2 (5.7%) | 2 (10.5%) | 0 | 99 (11.3%) |
| ≥1 ADR of special interest | 9 (1.2%) | 7 (1.1%) | 1 (2.9%) | 1 (5.3%) | 0 | 14 (1.5%) |

Off-label use included patients with low/medium DRL (n=866), patients without use of psychosocial support (N=144), patients without alcohol dependence (N=19), patients aged <18 years old (N=0). ADR, adverse drug reaction; ALAT, Alanine aminotransferase; ASAT, Aspartate aminotransferase; CNS, central nervous system; DRL drinking risk level
